# Acoustic Field Characterization of Medical Array Transducers Based on Unfocused Transmits and Single-Plane Hydrophone Measurements

**DOI:** 10.3390/s19040863

**Published:** 2019-02-19

**Authors:** Torben Marhenke, Sergio J. Sanabria, Bhaskara Rao Chintada, Roman Furrer, Jürg Neuenschwander, Orcun Goksel

**Affiliations:** 1 Computer-Assisted Applications in Medicine, ETH Zurich, 8092 Zurich, Switzerland; bhaskara.chintada@vision.ee.ethz.ch; 2 Institute of Dynamics and Vibration Research, Leibniz University Hannover, 30167 Hannover, Germany; 3 Institute of Diagnostic and Interventional Radiology, University Hospital Zurich, Rämistrasse 100, 8091 Zurich, Switzerland; 4 Swiss Federal Laboratories for Materials Science and Technology, Empa, 8600 Dübendorf, Switzerland; roman.furrer@empa.ch (R.F.); Juerg.neuen@sunrise.ch (J.N.)

**Keywords:** ultrasound, acoustic holography, hydrophone measurements, Rayleigh–Sommerfeld, medical transducers, near field, safety index, plane wave, elastography

## Abstract

Medical ultrasonic arrays are typically characterized in controlled water baths using measurements by a hydrophone, which can be translated with a positioning stage. Characterization of 3D acoustic fields conventionally requires measurements at each spatial location, which is tedious and time-consuming, and may be prohibitive given limitations of experimental setup (e.g., the bath and stage) and measurement equipment (i.e., the hydrophone). Moreover, with the development of new ultrasound sequences and modalities, multiple measurements are often required to characterize each imaging mode to ensure performance and clinical safety. Acoustic holography allows efficient characterization of source transducer fields based on single plane measurements. In this work, we explore the applicability of a re-radiation method based on the Rayleigh–Sommerfeld integral to medical imaging array characterization. We show that source fields can be reconstructed at single crystal level at wavelength resolution, based on far-field measurements. This is herein presented for three practical application scenarios: for identifying faulty transducer elements; for characterizing acoustic safety parameters in focused ultrasound sequences from 2D planar measurements; and for estimating arbitrary focused fields based on calibration from an unfocused sound field and software beamforming. The results experimentally show that the acquired pressure fields closely match those estimated using our technique.

## 1. Introduction

Characterization of medical transducers and the acoustic fields they generate is a common need and standard process, during most development stages of ultrasound equipment and imaging sequences. Performance of the ultrasound imaging sequences (resolution, sensitivity) may be affected by deviations of the transducer elements from the assumed pressure fields. Moreover, certain sequences and higher powers with focused transducers may cause damage to human body and therefore transducers and sequences are subject to stringent standards, such as NEMA UD3-2004 [1] in the United States and the international standard IEC 62359 [2]. Accordingly, the characterization of acoustic fields is a crucial step for certification of new ultrasound transducers. Regarding safety, mechanical index (MI) and thermal index (TI) are the two important parameters, where the former characterizes the possibility of cavitation and the latter any potential thermal damage to tissue [3]. TI estimates the increase of temperature that occurs in the ultrasound scanning region, with a TI of 1.0 meaning an expected temperature rise of 1° C. TI is defined as a worst-case estimate of the ratio of the power exposing the tissue during use with respect to the power required to cause a maximum temperature increase of 1 °C under identical scanner operating conditions [2]. MI defines the likelihood of reaching the onset of inertial cavitation according to [4]. For MI < 0.7 it is considered that physical conditions are unlikely to support bubble growth and collapse. Details of TI and MI calculation are provided in the Appendix A. For characterizing both above [2,4], the maximum pressure from the transmission in the field of view is an important measurement required.

With ultrasound arrays this is not a fixed value for each probe, but it also depends on the particular ultrasound sequence and transmit beamforming.

Most ultrasound sequences, such as beamforming and displacement tracking methods rely on simplifying assumptions, for instance, baffled piston transducer models or ray-tracing equations, see [5,6]. Such assumptions may lead to large deviations from the acoustic behavior of real transducers. For instance, for single crystal transducers, heterogeneous vibration patterns are observed on the transducer surfaces, and baffled piston models can introduce pressure deviations >50% (−6 dB) on the axial axis and completely fail to reproduce side lobes [7]. The general expression of a transmitted acoustic field including the location and the local expression of a focus point as well as any side-lobes, are typical questions relevant to transducer and imaging development. Additionally, for quality-control, testing the functionality and uniformity of all array elements becomes an important concern.

Characterization of acoustic fields is typically performed with temperature-controlled water bath experiments, where an acoustic recording device (“hydrophone”) is used on a mechanical translation stage to record the time-varying output at many locations of interest in space, while repeating the acoustic transmit at each point. Since this is a highly time-consuming process, often a single plane or a small focal area is scanned, and the testing of several transmission parameters becomes prohibitive, therefore most development currently being performed on simulation software.

A review of ultrasound modeling efforts for medical imaging is provided in [8]. State-of-the-art medical ultrasound simulators, for instance, Field II [9], Focus [10], and k-Wave [11], use simplified descriptions of medical ultrasound arrays, assuming a uniform vibration field over the elements (baffled piston models). The spatial projection of pressure fields from far field measurements to the transducer source plane could potentially provide more accurate descriptions of real imaging transducers. For heterogeneous media, the propagation domain needs to be fully discretized with finite-difference or finite-element methods, which lead to computationally demanding solutions.

For homogeneous media, an alternative to finite-difference or finite-element models is the direct holographic calculation approach. Several projection methods have been proposed, such as the Angular Spectrum Approach (ASA) in the Fourier domain [12] and Rayleigh integrals in the spatial domain [13]. A restriction of these methods is that they calculate the pressure fields in the linear regime. The holographic approach is adequate to simulate wave propagation in the linear regime as a substitute of water bath measurements, where, in order to avoid damage to the hydrophone, measurements are performed at low acoustic output levels [14,15]. In therapeutic acoustic applications, which use higher acoustic outputs, non-linear simulation models are required. However, state-of-the-art non-linear simulation models also utilize holography approaches to characterize the input transducer fields, which are then used as source function [13,16]. Therefore, acoustic holography is a general and efficient computational tool for transducer characterization.

Holography has been extensively used to characterize single-element large-area transducers, for which imperfections in the radiating surface are sought (for instance, [7,16,17,18,19]). Ghanem et al. [14], Kreider et al. [15] and Clement and Hynynen [20] applied holography to multi-element therapeutic arrays. In all these works, the characterized transducer elements are much larger than the imaging wavelength. Sapozhnikov et al. [13] applied holography to a convex imaging probe with crystal elements of wavelength size, however, the full-aperture was excited and individual element contributions were not investigated.

In this work we apply acoustic holography to characterize acoustic fields at single crystal level. We propose a spatial-domain re-radiation method to characterize pressure fields from a broadband hand-held medical imaging probe. We herein present results for a linear array consisting of a large number of elements (x128) and a relatively small footprint, each element being approximately of the wavelength size. Therefore, high-resolution near-field measurements for each separate element would be difficult to quantify. Instead, we explore the characterization of the full array behavior based on a single unfocused transmit. We experimentally validate our model for single element characterization, and both focused beamforming and non-focused plane wave ultrasound sequences. Based on a single unfocused plane wave excitation, the re-radiation method allows (i) to detect faulty elements in the transducer array, (ii) to simulate a focused sound fields, and (iii) to estimate safety indices. We also present the practical aspects of our experimental setup, e.g., to avoid fully immersing our ultrasound probe.

## 2. Materials and Methods

### 2.1. Numerical Reconstruction

For our re-radiation technique, we use the Rayleigh–Sommerfeld integral and time reversal acoustics for three-dimensional sound field reconstruction, based on the theory and equations given in [7,19,21,22,23]. We use a linear model of pressure field propagation, which allows us to build a holographic representation of the three-dimensional field in function of two-dimensional plane measurements. We summarize herein the main steps and building blocks relevant to our methodology. Wave propagation of acoustic pressure waves *p* in linear, incompressible fluids can be described with the Helmholtz equation

(1) $$\nabla^{2}p-\frac{\partial_{t}^{2}p}{c}=0$$

where *c* is the speed of sound and p(x,t) is an scalar field in space x=(x,y,z) and time *t*. p(x,t) is expressed in the Fourier domain as a superposition of harmonic plane wave fields:

(2) $$p\left(k,\omega \right)=\int_{-\infty }^{\infty }\int \;\;\;\int \;\;\;\int_{-\infty }^{\infty }p\left(x,t\right)e^{-i\left(\mathrm{kx}+\omega t\right)}dxdt$$

whereby ω is the harmonic component and k a vector of wave numbers. Transforming Equation (1) to the Fourier domain we obtain

(3) $$\nabla^{2}p+k^{2}\;p=0,\;k=\frac{\omega }{c}$$

**Figure 1:**
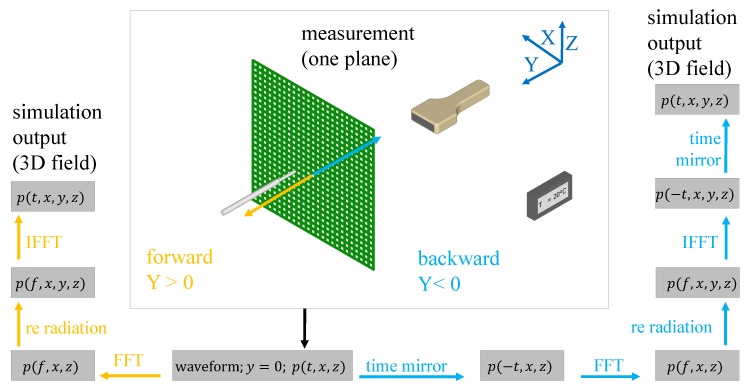
Implementation of the re-radiation method.

Substituting Equation (2) in Equation (3) reduces one degree of freedom in k, with $k_{y}={\left(k^{2}-k_{x}^{2}-k_{z}^{2}\right)}^{1/2}$, and allows expressing the three-dimensional pressure field in function of the pressure field at a single arbitrary plane y=0 (Figure 1)

(4) $$p\left(y,k_{y},k_{z},\omega \right)=\int_{-\infty }^{\infty }\int \;\;\;\int_{-\infty }^{\infty }p\left(x,\omega \right)e^{-i\left(k_{x}x+k_{z}z+\omega t\right)}e^{-ik_{y}y}dxdzdt=p\left(0,k_{x},k_{z},\omega \right)e^{-ik_{y}y}$$

The calculation of volumetric fields from single planes measurements allows a holographic representation of the pressure field. Equation (4) is transformed back to the spatial domain as:

(5) $$p\left(x,\omega \right)=\int_{-\infty }^{\infty }\int_{-\infty }^{\infty }p\left(\hat{x},0,\hat{z},\omega \right)g_{\mathrm{PS}}\left(x-\hat{x},y,z-\hat{z}\right)d\hat{x}d\hat{z}$$

where $g_{PS}$ is a spherical source convolution kernel

(6) $$g_{\mathrm{PS}}\left(x\right)=\frac{e^{-ik{∥x∥}_{2}^{2}}}{2\pi {∥x∥}_{2}^{2}}\left(\frac{1}{{∥x∥}_{2}^{2}}+ik\right)\frac{x}{{∥x∥}_{2}^{2}},$$

Equation (5) provides a holographic representation of the volumetric pressure field as a superposition of spherical waves distributed on an arbitrary plane y=0. This expression is a Rayleigh–Sommerfeld integral and an expression of Huygen’s principle [21]. In numerical computation, the integral Equation (5) is discretized with a finite measurement window *N* and pixel size *h*

(7) $$p\left(x,\omega \right)≅\sum_{s=1}^{N}h^{2}\;p\left({\hat{x}}_{s},0,{\hat{z}}_{s},\omega \right)g_{\mathrm{PS}}\left(x-{\hat{x}}_{s},y,z-{\hat{z}}_{s}\right)$$

where ${\hat{x}}_{s}$ and ${\hat{z}}_{s}$ are the measurement plane coordinates.

A complete derivation with intermediate steps and additional information can be found in [7,24].

### 2.2. Proposed Technique

An overview summary of our proposed technique is shown in Figure 1. The measured (calibration) acoustic field data (herein, in *x*-*z* plane) are used after the measurements as input for sound field reconstruction by re-radiation. The results of the three-dimensional reconstruction p(x,y,z,t) are then validated with experimentally acquired acoustic field data at the same spatial positions.

In the first step, the time waveforms at each measured point p(x,z,t) are converted to the spectral domain p(x,z,f) with a Fast Fourier Transform (FFT). For the forward re-radiation, the sum of Equation (7) is calculated after the transformation. In the last step, inverse FFT (IFFT) is used to convert the frequency-dependent fields p(x,y,z,f) back to the time domain p(x,y,z,t). Backward re-radiation is also performed similarly, by adding two steps: Both before the FFT step and again after the IFFT step, the time waveforms are mirrored in time, i.e., p(x,y,z,−t). Both calculation methods are used in this work. The backward re-radiation calculates the sound pressure distribution in the region between measuring plane and transducer. The forward method is applied for sound fields outside this region. In order to better demonstrate our technique, we present our method below on an actual setup, which is also the experimental configuration that we later use in our data evaluation.

### 2.3. Materials

**Figure 2:**
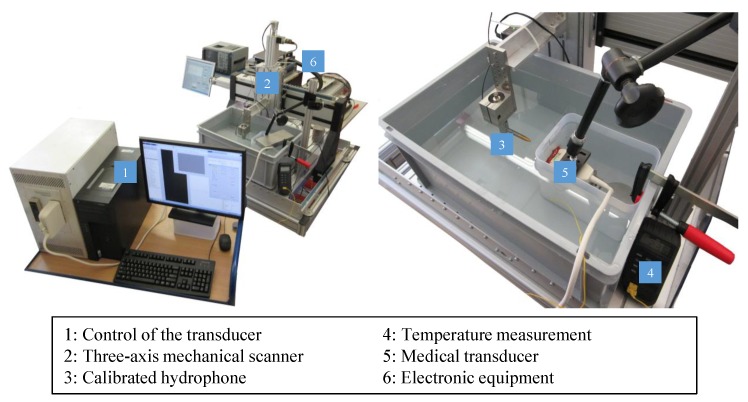
Hydrophone measurement setup.

Our experimental test bench setup for wave-field characterization is shown in Figure 2.

The sound waves emitted by a phased array transducer (Tx, Pos. 5) are measured by means of a receiver (Rx, Pos. 3), which is attached to a three-dimensional scanner (Pos. 2). This structure makes it possible to measure the position and time dependent sound field in high resolution. We implemented our custom transmit (Tx) sequences on a Verasonics Vantage 256 system (Verasonics, Redmond, WA, USA) for controlling individual elements of an ATL L7-4 Linear probe (ATL Philips, Bothell, WA, USA) to achieve the desired modes of data acquisition. This linear probe has 128 elements at a pitch of 0.3 mm, and an operational frequency range of 4–7 MHz, with 5 MHz being the center frequency.

In order to prevent the transducer from being permanently held under water, which could lead to ingress of water into the housing by the water pressure and thus to transducer damage, only the radiation surface was in contact with water. For this purpose, the transducer was placed in a plastic box, which was clamped and fixed with weights in order to prevent floating and drift. The transducer has a free radiation surface through a window cut on the side of this box, where the gap between the box and the transducer was sealed with silicone glue (Elastosil E43, Wacker Chemie AG, Munich, Germany). In order to avoid residues of this adhesive on the transducer, the latter was previously covered with insulating tape, which could be easily removed later without any damage to the transducer. The radiation surface was in direct contact with water, with no contact to tape or adhesive. To fix the transducer, a flexible clamp with swivel joints was used. We did not wrap the transducer with a plastic bag, in order to avoid possible air bubbles that could be trapped between the transducer and the bag as well as any false signals from potential acoustic impedance changes on the bag surface. In addition, this design prevents the entire transducer from having to be immersed in water, which is not possible with many transmitters due to the non-watertight housing.

As receiver, we used a calibrated hydrophone Rx (HGL-0200, ONDA, Sunnyvale, CA, USA) with nominal sensitivity of 50 nV/Pa and a capacitance of 30 pF. The diameter of the electrode aperture is 0.2 mm, which results in a frequency range (±3 dB) of 0.25–40 MHz. At the examined frequency of 5 MHz the acceptance angle (−6 dB) is 100°, thus providing a quasi-isotropic receive response. A preamplifier (AG-2010, ONDA), a DC block (AH-2010-DCBNS, ONDA) and a termination impedance (1-1478207-0, Tyco Electronics, Schaffhausen, Switzerland) are needed for signal amplification and processing.

For high-precision measurement of the three-dimensional sound pressure distribution, the hydrophone was attached to a motorized scanning system with three linear scanning axes (ISEL Germany AG, Eichenzell, Germany). The X, Y, and Z axes were three ball screw feed linear axes with working distances of 500, 500, and 170 mm, respectively, and with a positioning reproducibility of 0.01 mm. To synchronize this scanning system and the excitation and acquisition of transducer signals, a software in LabVIEW^®^ (National Instruments Corp., Austin, TX, USA) was written [25]. The coordinate system of the scanning unit was aligned with the housing of the transducer.

The container was then filled with water as the acoustic propagation medium. To prevent impurities or air bubbles in the water from having an influence on the result of the measurement, distilled water was used. The water was enriched with silver ions to prevent algae formation [26], in order to have constant conditions in the water over the 5 days time period, wherein all measurements were acquired. The sound speed in the propagation medium is an important input parameter for the re-radiation method, as seen in Section 2.1. Since sound speed changes significantly with temperature, the water temperature was measured using a thermometer (DTM-307, Tecpel, Taipei, Taiwan), with a resolution of 0.1° and an accuracy of ±0.3%.

The grid steps of the measured *x*, *z* input planes for the re-radiation were roughly defined as λ/3≈0.1 mm for $\lambda =c/f=\frac{1491\;m/s}{5\;\mathrm{MHz}}\approx 0.3$ mm. The window size *N* was adapted individually for each measurement depending on the sound pressure distribution and the size of the sound field. For example, larger areas must be measured for unfocused sound fields. Based on the measurement results, an empirical distance threshold for a −30 dB drop could be defined.

### 2.4. Application Scenarios

With the re-radiation method, it is possible to reconstruct both time-dependent data (A, B-scans) as well as amplitude distributions (C-scans) as in [24]. We focus here on peak amplitude characterization, since it both summarizes power profiles and coherence of the ultrasound field. Low acoustic outputs are used in the ultrasound probe to avoid damage to the hydrophone. Due to the high sensitivity of the hydrophone ($5\times 10^{-8}\;V/\mathrm{Pa}$), signal-to-noise ratio above 60 dB is achieved for all measurements.

**Figure 3:**
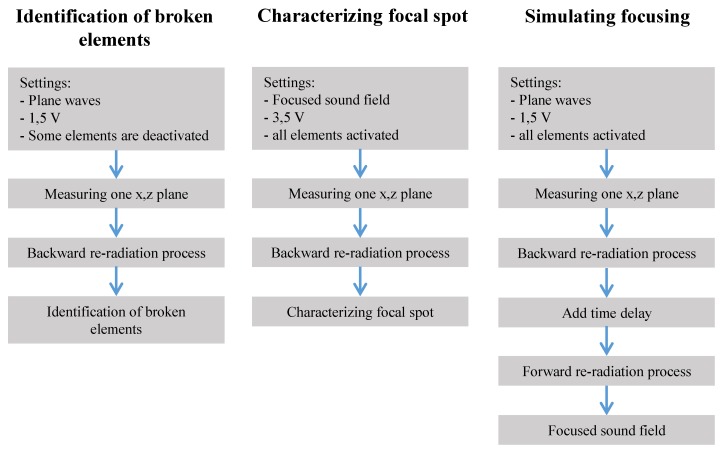
Performed examinations and simulations.

Our goal herein to characterize the acoustic field and the transducer with as little effort (measurement) as possible. Three example application scenarios are illustrated in Figure 3 and explained in the result section.

## 3. Results and Discussion

In the validation, amplitude images (C-scans) were generated by recording the peak value of the time waveform at each scan position, which was visualized as 3D volumetric plots, 2D cross-sections or 1D profiles. The pressure images are represented in logarithmic scale normalized with respect to the maximum pressure, and errors between reconstruction $\hat{p_{l}}$ and measurement $p_{i}$ are plotted in the same logarithmic scale.

The maximum pressure deviation (maxDev) was calculated as:

(8) $$\mathrm{maxDev}=\max\frac{\left|p_{i}-\hat{p_{l}}\right|}{p_{max}}$$

Since measurements are performed on a discrete spatial grid, in order to remove discretization uncertainties originating from any potential sub-pixel mis-registration, a 3-by-3 median filtering was applied to the C-scans $p_{i}-\hat{p_{l}}$ before calculating MaxDev. The Matlab^®^ software (The Mathworks Inc., Natick, MA, USA) was used for all calculations.

### 3.1. Case 1: Identification of Faulty Elements

**Figure 4:**
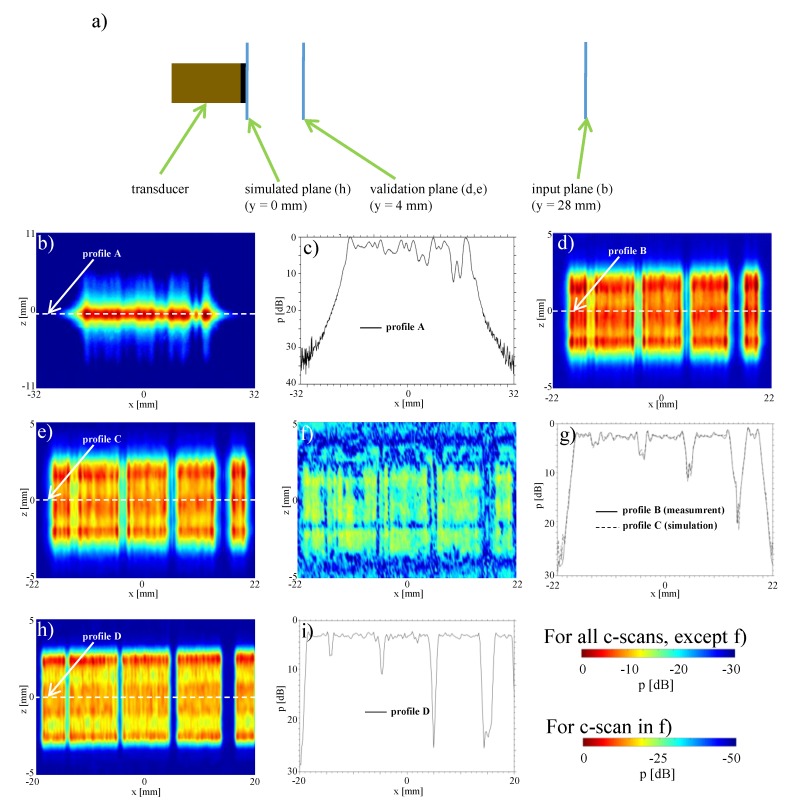
Identification of broken elements: (**a**) setup; (**b**) measurement of the pressure distribution in a x,z-plane 28 mm in front of the transducer; (**c**) measurement profile y=28 mm, z=0 mm; (**d**) measured sound field 4 mm in front of transducer (y=4 mm); (**e**) simulated sound field 4 mm in front of transducer (y=4 mm); (**f**) difference (c-scan) between measurement and simulation 4 mm in front of transducer (y=4 mm); (**g**) difference profile between measurement and simulation 4 mm in front of transducer (y=4 mm, z=0 mm); (**h**) simulated sound field directly in front of transducer (y=0 mm); (**i**) amplitude profile directly in front of the transducer y=0.

To simulate faulty elements in the array, we herein deactivated some elements of an otherwise functional array. In order to study sensitivity to detect a broken aperture, consecutive elements in groups of one, two, four, and eight elements were deactivated; in particular, herein the element indices corresponding to 16, 47–48, 78–81, and 109–116. For these settings Figure 4b shows the *x*-*z* plane measurement of the sound pressure distribution at a distance of y=28 mm from the transducer surface, while transmitting a plane-wave unfocused field by firing all transducer elements simultaneously.

The failure of eight elements can be seen clearly in the two-dimensional C-scan Figure 4b, as well as in the profile in Figure 4c with an amplitude decrease of ≈10 dB. Similarly the four and two elements are still visible, but the amplitude drop becoming only 7.5 dB and 5 dB, respectively. A frequent occurrence in practice is the failure of a single element. This more realistic scenario can not be identified in our results in the 2D image, nor in the profile given, as the signal drop is in the range of acoustic fluctuations. In all cases above, it is also difficult to impossible to precisely identify the actual numbers of broken elements in each region.

For evaluation, we carried out measurements as close as possible to the transducer surface, which are typically avoided in practice especially at high amplitudes to prevent hydrophone damage. We measured the *x*-*z* plane at y=4 mm distance from transducer surface, as seen in Figure 4d. This was then compared to the reconstruction of acoustic pressure computed via sound field re-radiation (back-propagation) to that plane from the y=28 mm measurement above (Figure 4e). The reconstruction and the measurements visually show a very good agreement, where a map of spatial absolute differences is shown in Figure 4f. The maximum error (maxDev) is in the range of −20 dB. To demonstrate the agreement, a comparison of both profiles at (y,z)=(0,0) is shown in Figure 4g. Amplitude deviations both in the deactivated areas as well as the emission areas and at edges are very well represented by the reconstruction.

With our re-radiation method, the sound pressure distribution can also be determined directly on the surface of the transducer, which cannot be measured with a hydrophone due to risk of its collision with transducer surface. The reconstructed field at y=0 mm based on the measured sound field at y=28 mm is shown in Figure 4h. When compared to the C-scan at y=4 mm, the edges of the deactivated areas can be seen even more clearly, with the number of “dead” elements clearly identifiable. This is further illustrated by the profile shown in Figure 4i. The amplitude drops at the deactivated elements are even more pronounced than those at y=4 mm.

Table 1 summarizes the maximum and average amplitude drops. It can be seen that the amplitude drop in the profile on the transducer surface at y=0 is larger than that of y=4 mm in front of it. From these profiles, it is also possible to make accurate statements about the number and position of faulty elements, thanks to sharp edges. It is also possible to some extent to identify variations in transmission efficiency between transducer elements. For one element deactivated there is cross-link of adjacent channels with −6.4 dB. When considering two and four elements simultaneously, cross-channel interference is reduced to −11.1 dB and −26.5 dB, respectively. Therefore, by using the given re-radiation method, it is therefore possible to draw conclusions about the state of individual array elements. We can also identify pressure variations for single transducer elements over the elevation plane.

**Figure 5:**
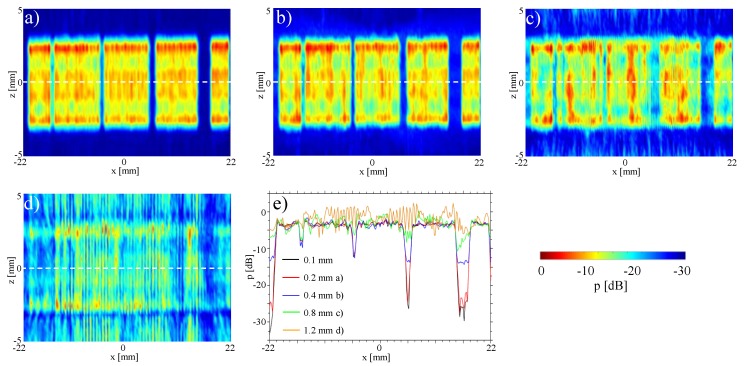
Analysis of the influence of different resolutions of the input area on the re-radiation result: (**a**) 0.2 mm; (**b**) 0.4 mm; (**c**) 0.8 mm; (**d**) 1.2 mm; (**e**) Comparison.

We further investigate the influence of the grid step of the input measurement planes in the reconstruction of the pressure fields at individual array elements (Figure 5). The pressure field distributions at the single deactivated element are consistent for grid steps down to 0.4 mm (Figure 5a,b), with degradation observed above this value (Figure 5c,d). These results suggest that a grid step of the wavelength is sufficient for reconstruction. Remarkably, we are able to reconstruct the sound field of elements of approximately the wavelength size with coarser grids. This shows that the holography method is able to decode near-field information from far-field measurements.

### 3.2. Case 2: Characterizing a Focal Spot

Similarly to the above example with an unfocused transmission, the method can also be used for the computation of focused sound fields. This is relevant, for example, to characterize the maximum pressure at the focus, required for safety regulations in the certification of imaging equipment and sequences. Typically, the maximum pressure location is sought by a time-consuming procedure of repeated measurements of different planes using a hydrophone. We herein demonstrate re-radiation as an alternative method measurement only on a single plane.

**Figure 6:**
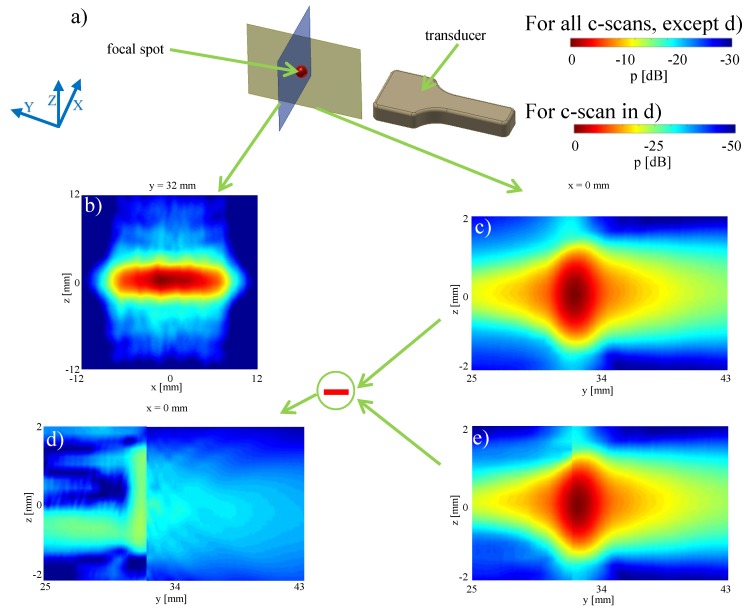
Characterizing the focal spot: (**a**) setup; (**b**) x,z plane measurement 32 mm in front of the transducer; (**c**) y,z plane measurement in the middle of the transducer (x=0 mm); (**d**) difference between the measured and simulation sound field; (**e**) simulation of the pressure distribution around the focal spot based on the re-radiation method.

For this purpose, we programmed an imaging transmit sequence focused at a focal distance of 32 mm, with a pulse length of five periods, i.e., 5/f=1 μs. The focal spot at y=32 mm was chosen to reproduce a realistic ultrasound imaging configuration. The focal spot at ultrasound imaging configurations is typically located at the center depth of the field of view to achieve optimal focusing over the full region of interest [3]. Thus a focus at y=32 mm is appropriate for a measurement window depth of 65 mm, which is roughly the maximum imaging depth in human soft tissue for the L7-4 probe. From a measurement of the x=0 plane, the maximum pressure was identified to be at depth y=32 mm, at which plane the sound field was also measured as seen in Figure 6b.

Starting from this plane measurements, the acoustic field was then re-radiated backward (cf. Figure 6e). The absolute difference of this reconstruction from actual measurements, see Figure 6c, in the x=0 plane is shown in Figure 6d. The simulated cross-section in Figure 6e shows a high level of detail and the shape of the sound field is seen to be well reproduced. The maximum error in comparison between measurement and reconstruction (maxDev) is −22.74 dB ≈ 7.29% of the maximum pressure.

**Figure 7:**
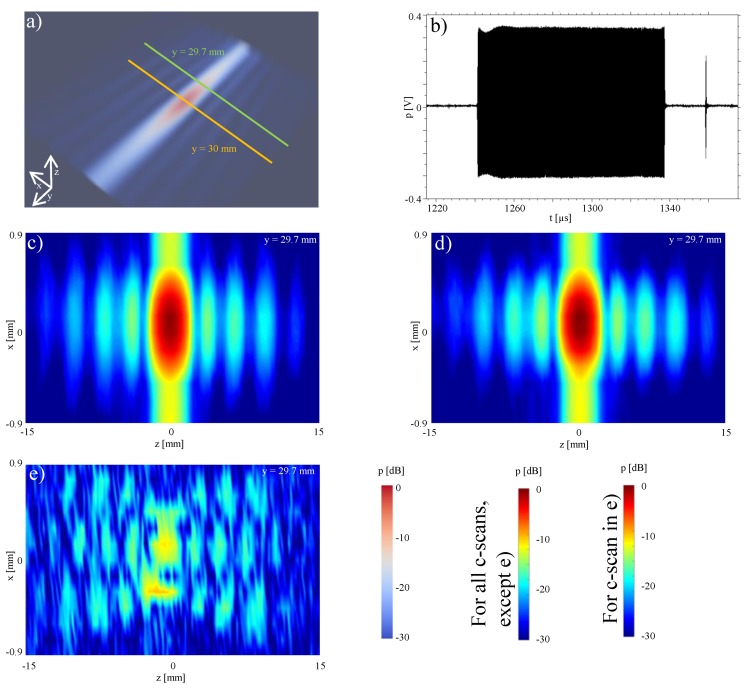
Characterization of an ultrasonic push: (**a**) 3-dimensional hydrophone measurement of a ultrasonic push; (**b**) time waveform of a push; (**c**) x,z-plane measurement; (**d**) x,z-plane re-radiation; (**e**) difference between measurement and simulation.

For illustration of the applicability of the method to longer ultrasound pulses, the reconstruction for the focal point was also performed for an ultrasonic push for acoustic-radiation force used for shear-wave elastography. We used a pulse time length of ≈100 μs as seen in Figure 7b between 1240 and 1340 μs. The ultrasonic push induces an acoustic radiation force into the tissue and generates a shear wavefront, which propagates slowly with speeds 1–10 m/s. In order to track this shear wavefront, additional tracking beams are generated after the shear wave push, the first of which is visible in Figure 7b at 1380 μs. These are conventional imaging beams (similar to the focused beam in Figure 6, which are in used to track the shear wavefront. The tracking beams are used to measure the speed of the shear wavefront, from which the mechanical properties of the tissue are calculated [27,28].

A three-dimensional measurement of the pressure field of the ultrasound push around the focus point is shown in Figure 7a, in a region of interest of 2 × 20 × 20 mm${}^{3}$. Starting from and using only the measurements at y=30 mm plane, the sound pressure distribution in this region was numerically calculated using backward re-radiation. The results (see Figure 7d) exhibits a sufficiently good agreement with the measurements (see Figure 7c). The difference plot in Figure 7e (maxDev of −18 dB) demonstrates that the presented method can also be used for complex transmission with longer pulses. Based on Figure 6 and Figure 7, we cannot conclude an association between longer signals and larger maxDev, which is also not supported by the literature (e.g., [29]). We hypothesize that the differences observed between Figure 6 and Figure 7 are related to the larger field spread in the measurement plane for Figure 7c with respect to Figure 6b. At the boundaries of the available measurement window of Figure 7c, relatively large pressure values (−15 dB) are observed. Due to the finite measurement window size, discretization uncertainties are introduced in the re-radiation process. The effect of finite window size has been studied in detail in [7], where maxDev is of the same order of magnitude as the pressure level at the boundaries of the measurement window. This is similar to observations of the analyzed re-radiation configurations herein.

A common denominator of quasi-continuous wave fields as in Figure 7 and off-axis focused configurations (here not covered), is the presence of larger sidelobes with respect to pulsed axial focusing (e.g., Figure 6b) [3]. Figure 7c, shows that our method is able to correctly reproduce the pressure field of the main lobe (maxDev = −18 dB), but also of the sidelobes, with maxDeV = −28.8 dB, −30.5 dB and −32.7 dB for the second, third and fourth lobes, respectively.

### 3.3. Case 3: Simulating Arbitrary Beamforming from Unfocused Transmit

Ultrasound sequence design and development is often carried out on simulation software, with synthetic transducer definitions. Actual measurements can give a better transducer representation. Using our method, one can estimate transducer surface radiation for a particular transducer, which can then be re-radiated with arbitrary time-delays synthetically applied at each crystal in beforehand to simulate any transmit sequences. This allows for both accurate and realistically affordable development cycles of ultrasound sequences. To demonstrate this, we herein take measurements from an unfocused (plane-wave) transmit, based on which we simulate a focused sound field.

**Figure 8:**
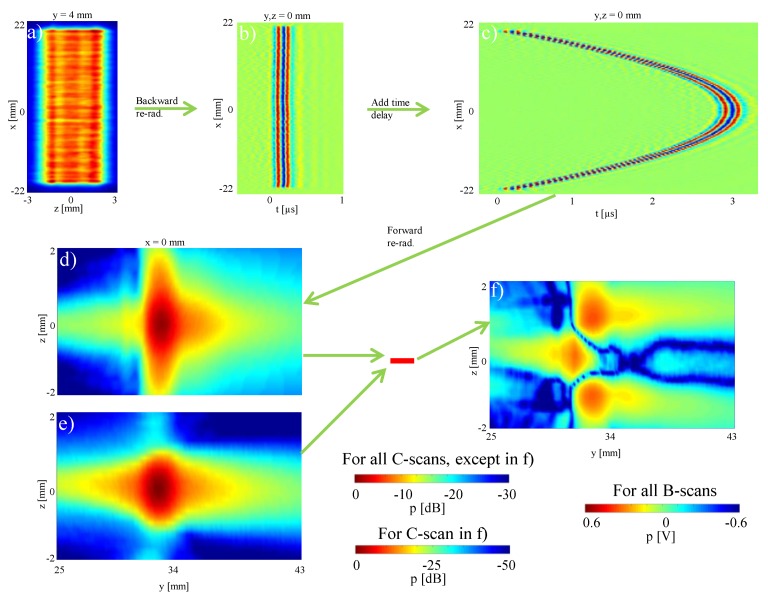
Simulated focusing: (**a**) measured pressure distribution 4 mm in front of the transducer; (**b**) B-scan result of the backward re-radiation to the transducer surface plane; (**c**) B-scan with an added time delay for a focus length of 30 mm; (**d**) simulated, focused sound field based on the forward re-radiation method; (**e**) measured sound field around the focal spot; (**f**) difference between the simulated and measured sound field.

Similarly to Case 1, but without simulating faulty elements, we took a measurement at y=4 mm, as shown in Figure 8a.

We then backward re-radiate the pressure field measurement at y=4 mm to determine the sound pressure distribution on the transducer surface at y=0. The B-scan time-signals at (y,z)=(0,0) can be seen in Figure 8b, confirming an unfocused plane-wave. We then added time delays to this signal in order to simulate a focused transmit at a distance of y=30 mm. These time delays were calculated using an empirical formula based on the geometrical relationship as follows

(9) $$t_{i}=\frac{\sqrt{z_{f}^{2}+d^{2}}-\sqrt{z_{f}^{2}}}{c},$$

where *d* is the lateral distance, i.e., in *x*, from the transducer center and $z_{f}$ represents the focusing distance [30,31,32]. The simulated focused transducer signal with this simple software transmit beamforming process is shown in Figure 8c. Providing this as input to forward re-radiation from transducer surface, the sound pressure distribution in the area of the focus point was calculated. For example, in Figure 8d a section of the x=0 plane is shown. For validation, this was compared with the measured focused sound field at that distance, cf. Figure 8e. Their absolute difference is shown in Figure 8f. It can be seen that the inner area of the focus point (between z=−0.2 mm to z=0.2 mm) is very well represented by the simulation. The comparison of the maximum sound pressure of the measurement and the simulation in the focal spot shows deviation of −41.9 dB (0.8 %), which is quite small and in good accordance with holographic literature, e.g., [13]. In the area around the focal point, higher sound amplitudes are simulated. The maxDev value for the whole simulated sound field is −10.1 dB. As Figure 6 shows, the y-scanning direction and thus the focusing direction was not completely aligned with the propagation direction of the ultrasonic waves in our experimental setup. This is visible in Figure 6c by the slightly asymmetric pressure field distribution along the vertical axis (*z*), which shows that the transducer radiation axis is not perfectly parallel to the horizontal axis (*y*) of the measurement window. Although the housing of the transducer was aligned carefully, it is not possible to guarantee that the elements are perfectly parallel to the housing and thus the waves are emitted parallel to the external transducer housing. Despite these small differences in the dimension of the focal point, the simulation shows that the area of the location and the amplitude of the maximum pressure, which are most essential parameters, are accurately estimated.

An advantage of this reconstruction method is that the maximum sound pressure can be determined as a function of the focus distance without additional experimental effort. Thus, it is possible to determine the overall maximum sound pressure for a complete characterization. A further advantage is, that sound pressures from focused sound fields that are outside the measuring range of hydrophones can be determined. In addition, our method provides the opportunity to simulate the propagation in other media and to determine the position of the focus point in a different material, e.g., human soft tissue.

### 3.4. Determination of Ultrasonic Safety Measures

We herein calculated and evaluated MI and TI estimation from re-radiation in comparison to those from measurements. The calculation procedure is detailed in the Appendix A. The following pressure values were used as measured input values for MI and TI calculations:

1. Direct measurement of the maximum sound pressure of the focused sound field using a hydrophone
2. Measurement of the sound pressure in a plane outside the focus point and calculation of the maximum pressure using the re-radiation method (see Section 3.2)
3. Measurement of the sound pressure in a plane of an unfocused sound field, re-radiation to calculate the pressure distribution at the crystals, and addition of delay law with Equation (9) on the calculated crystal pressure fields, from which the maximum pressure at an arbitrary focal point (same as in Section 3.2) is calculated with the re-radiation method (see Section 3.3).

The results of the MI and TI calculations are shown in Table 2.

The results show that both values can be reproduced by using the re-radiation method. Especially the simulated focusing (iii) from unfocused transmits shows very small deviations of 0.8% for MI and 1.6% for TI. Larger differences between the measurement and the simulated focusing from focused transmit (ii) with 4.4% for MI and 8.6% for TI may be due to the fact that the measured input plane and the calculated focus point were located at approximately the same plane. As a result, some continuity assumptions on which the re-radiation procedure is based on are only partially fulfilled; therefore leading to potential deviations in the results. Particularly, while Equation (5) is a continuous integral, the practical numerical implementation uses a discrete representation (Equation (7)). This has previously been observed in [7] to introduce artifacts in the holographic reconstructions in a ±5λ distance range from the measurement plane. This is here visible in Figure 6d, where an increase in the pressure deviations is observed close to the measurement plane (y=30 mm). A larger distance between the measuring plane and the focus point, as seen with (iii), leads to a reduction of the deviations. This results illustrate that a single unfocused field measurement is sufficient to characterize MI and TI for focused configurations, which further simplifies the transducer characterization effort.

## 4. Conclusions

Herein, we have presented the feasibility of fully characterizing pressure fields from medical transducer arrays, based solely on measurements on a plane aligned parallel to an array transducer surface. This implies that the transducer can be examined for faulty elements, the sound pressure distribution can be determined directly at the transducer surface, and the focus point can be reconstructed three-dimensionally to allow for determining the position and the amplitude of maximum pressure.

We have shown that the fields of individual transducer elements can be reconstructed at wavelength resolution from unfocused transmit single-plane far-field measurements. This also provides information about the homogeneity of single crystal fields over the transducer elevation plane. The input grid can be slightly coarser than the reconstructed wavelength, however, with grid step twice the wavelength reconstruction degradation is observed.

The characterization of maximum pressure radiated at the focal spot is also more accurate by simulating the beam-forming process based on the reconstructed crystal fields, rather than attempting to re-radiate the already focused fields acquired nearby the focal spot. The method shows a good agreement with experimental measurements for both pulsed and longer continuous wave sequences.

Characterization of the focal point based on the unfocused fields of individual elements, as illustrated in Figure 8, provides a high amount of flexibility for testing different imaging and beamforming sequences with a single measurement effort. In this work we show the feasibility of acoustic holography to reproduced representative ultrasound imaging configurations. With this we have covered typical pressure field distributions for focused and non-focused transducers, and pulsed-wave and quasi-continuous wave excitations. A systematic error analysis in function of beamforming parameters (for instance, focus depth, steering angle or apodization) was beyond the focus of this work, and shall be of interest in future studies.

For high-power sequences non-linear models are necessary for pressure field simulation, which are more computationally intensive. High-power measurements are also directly challenging to acquire without damaging the hydrophone. The proposed approach provides pressure distributions at the individual crystal elements, which can then be used as source functions for state-of-the-art non-linear models (for instance [13,16]). Therefore, its application is general for ultrasound transducer characterization.

## Figures and Tables

**Table 1:** Amplitude drops.

Deactivated Elements	4 mm		0 mm	
	Average Drop	Max Drop	Average Drop	Max Drop
1	−3.2 dB	−4.8 dB	−5.7 dB	−6.4 dB
2	−6.1 dB	−7.7 dB	−10.5 dB	−11.1 dB
4	−8.2 dB	−14 dB	−17.4 dB	−26.5 dB
8	−14.5 dB	−26.9 dB	−22.4 dB	−26.1 dB

**Table 2:** Calculation of MI and TI based on measured and calculated pressure values.

Index	(i) Measurement	(ii) Re-Radiation with Focused Transmit	(iii) Re-Radiation with Unfocused Transmit
MI (error%)	0.2818	0.2614 (7.3%)	0.2840 (0.8%)
TI (error%)	1.328	1.213 (8.6%)	1.349 (1.6%)

## Abbreviations

The following abbreviations are used in this manuscript:

ASA	Angular Spectrum Approach
FFT	Fast Fourier Transform
IFFT	Inverse FFT
MI	Mechanical Index
PNP	Peak Negative Pressure
Rx	Receiver
TI	Thermal Index
TIS	Soft Tissue TI
Tx	Transmitter

## Appendix A. Estimation of Mechanical and Thermal Indices

For medical transducers, mechanical and thermal indices must be determined and confirmed to be below certain limits, defined by international standards. The mechanical index (MI) estimates the likelihood of acoustic cavitation and consequent tissue damage, and is defined as [2,4]:

(A1) $$\mathrm{MI}=\frac{\mathrm{PNP}}{\sqrt{f_{c}}},$$

where PNP (MPa) is the peak negative pressure of the ultrasound wave, derated by 0.3 dB cm${}^{-1}$ MHz${}^{-1}$ in order to take into account the difference between the acoustic attenuation in tissue and water. $f_{c}$ (MHz) describes the center frequency, which is 5 MHz in our experiments.

Thermal index (TI) estimates potential risk of thermal bioeffects and damage. Given IEC 62359 [2], the experiments of this paper fall under the category of “non-scanning mode” for soft tissue at surface and soft tissue at focus. Accordingly, for apertures larger than 1 cm${}^{-2}$, TI is given by IEC 62359 [2] as:

(A2) $$\mathrm{TI}=\max_{z>1.5\;D_{\mathrm{eq}}}\left[\min\left[\frac{W\left(z\right)f_{c}}{210\;\mathrm{mW}\;\mathrm{MHz}},\frac{I_{\mathrm{SPTA}}\left(z\right)f_{c}}{210\;\mathrm{mW}\;{\mathrm{cm}}^{-2}\;\mathrm{MHz}}\right]\right],$$

where *W* and $I_{\mathrm{SPTA}}$ are the derated output power and spatial-peak temporal-average (SPTA) intensity, respectively, at a distance of *z* from the transducer surface. $D_{\mathrm{eq}}$ is the equivalent aperture diameter, depending on the total transducer surface area $A_{\mathrm{trans}}$ and is calculated by

(A3) $$D_{\mathrm{eq}}=\sqrt{\frac{4}{\pi }A_{\mathrm{trans}}}.$$

A $D_{\mathrm{eq}}$ of 0.0221 m is calculated by consideration the transducer edge lengths of 38.4 and 10 mm. The output power is calculated by

(A4) $$W=\frac{A_{f}{\mathrm{PNP}}^{2}}{\rho c},$$

ρ is density, *c* the speed of sound in the propagation medium, and $A_{f}$ the total surface area of the ultrasound illumination. The area depends on the focal distance $z_{f}$, the wavelength λ, and the transducer surface area $A_{\mathrm{trans}}$ and it can be approximated by the size of the focusing spot as

(A5) $$A_{f}=\frac{{\left(z_{f}\lambda \right)}^{2}}{A_{\mathrm{trans}}}.$$

At a wavelength of 0.3 mm and a focus distance of § mm, $A_{f}$ assumes a value of $2.09\times 10^{-7}\;m^{2}$. The spatial-peak temporal-average intensity $I_{\mathrm{SPTA}}$ depends on the output power *W*, the surface area of the ultrasound illumination $A_{f}$, and the total time of effective insonification $t_{\mathrm{inso}}$ as well as the total time of sonification $t_{\mathrm{so}}$ as follows

(A6) $$I_{\mathrm{SPTA}}=\frac{W}{A_{f}}\cdot \frac{t_{\mathrm{inso}}}{t_{\mathrm{so}}}.$$

The limit values of the coefficients depend on the boundary conditions. For fetus, for example, the limits are lower than for adults. The very highest limits allowed by the standard are 6.0 for TI and 1.9 for MI.
